# Exploring agency, communion and narrative foreclosure in cognitive behavioural therapy for substance use disorders

**DOI:** 10.1016/j.abrep.2025.100626

**Published:** 2025-07-24

**Authors:** Mark de Lange, Job van der Palen, Hein de Haan

**Affiliations:** a Tactus Addiction Treatment, Deventer, The Netherlands; b Present: Trajectum, Zwolle, The Netherlands; c Department of Epidemiology, Medisch Spectrum Twente, Enschede, The Netherlands; d Section Cognition, Data and Education, Faculty of Behavioural, Management and Social Sciences, University of Twente, Enschede, The Netherlands

**Keywords:** Addiction, Agency, Communion, Foreclosure, Narrative identity

## Abstract

**Introduction:**

Substance use disorders (SUDs) are a significant societal concern, negatively impacting self-worth, hope, and interpersonal connectedness. SUD recovery involves a transformation of one’s ‘life-story’ or ‘narrative identity’. ‘Narrative foreclosure’ (NF) impedes this process by disconnecting past, present, and future narratives, hindering the rewriting of SUD-related identities. We investigate the impact of SUD-based cognitive behavioural therapy (CBT) on narrative identity transformation and hypothesise NF as a potential factor in post-treatment relapse.

**Methods:**

107 patients referred to an outpatient clinic for adults with SUDs in the Netherlands were included. Participants completed the “Agency and Communion Inventory” (translated and validated in Dutch), the Narrative Foreclosure Scale (NFS) and the “Measurement of Addiction for Triage and Evaluation” (MATE), including the Depression, Anxiety, and Stress-Scale (DASS-21). Data collection occurred at the start of treatment, post-treatment (+3 months), and during a follow-up measure (+6 months).

**Results:**

While participants showed a substantial reduction in substance use frequency and DASS-21 sum scores during CBT-treatment, no such differences were found in narrative themes. Multivariate models revealed that baseline communion and past-directed NF predicted ’substance-use reduction’ during treatment, while ’cannabis use,’ communion and future directed NF were predictors of ’DASS-21 sum score reduction’. Baseline communion showed a moderate positive relation to post-treatment relapse, while ’NFS: Future’ exhibited a moderate inverse relation. These findings remained robust even when altering the criteria for defining ’post-treatment relapse.’ They indicate that a stronger identification with communal themes serve as a protective factor against relapse, while future directed NF is associated with higher relapse rates.

**Conclusion:**

this study provides valuable insights into the interplay between narrative themes and treatment outcome(s) in SUD recovery. Notably, our findings underscore the significance of future-directed narrative foreclosure in the recovery process and found that CBT for SUDs does not appear to significantly facilitate narrative identity transformation. These results suggest that existing addiction treatments could be enhanced through the integration of narrative-focused interventions.

## Highlights

• Cognitive behavioural therapy didn’t impact narrative themes.
• Communion and future-related narrative foreclosure relate to short-term outcomes.
• A closed-off view of the future may make relapse more likely post-treatment.
• Agency didn’t predict outcomes, contrasting prior research on its role in SUD recovery.
• Integrate narrative-focused techniques to address foreclosure and enhance communion.

## 1 Introduction

Substance use disorders (SUDs) are an ongoing concern for society and the high prevalence and relapse of substance use disorders continue to challenge the field (Bowen et al., 2014). SUDs negatively impact a patient’s self-worth (Van Erp and Van der Meulen, 2017, Van Boekel, 2015), hope, self-esteem, sense of personal empowerment (Livingston & Boyd, 2010) and one’s sense of belonging or ‘interpersonal connectedness’ (Chou et al., 2011). SUD-patients may also experience a discrepancy between the person they have become and who they believed themselves to be ‘at heart’, before substance use (McIntosh, 2002). This discrepancy and resulting ‘identity fragmentation’ (e.g. Singer, 2013, Singer et al., 2013) negatively impacts a patient’s other social identities (e.g. the self as partner, parent or employee) (McIntosh & McKeganey, 2000). SUD-recovery therefore, is fueled by a movement from a patient’s SUD-related ‘social identity’ (how one views oneself within the context of specific roles: e.g. the self as ‘substance user’), to a ‘recovery identity’ (Dingle et al., 2015, McIntosh, 2002). This transformation is associated with an increased identification with two core narrative themes that organise personal meaning-making within the life story: agency (a focus on autonomy, competence, and self-assertion) and communion (a focus on connection, belonging, and care) (McConnell and Snoek, 2018, McWeegman, 2010, Singer, 2013, Singer et al., 2013, Rowlands et al., 2019). These themes reflect fundamental motivational orientations that frequently recur in life narratives and help shape one’s evolving sense of self over time (McAdams, 2018).

### 1.1 Narrative approach

In narrative psychology, one’s identity is represented as an internalised and evolving life-story: a self-narrative that integrates life across time and provides coherence, meaning, and purpose (e.g. Crossley, 2000, McAdams, 2003, McAdams, 2018). McAdams (2018) refers to this construct as one’s narrative identity — a generalised story about “how the I came to be the person the I is becoming.”

According to Dunlop (2017), individuals develop multiple narrative strands, each tied to a specific social context or role − such as the self as partner, employee, or patient. These social identities represent how individuals perceive themselves in relation to others or to specific roles and groups (McIntosh & McKeganey, 2000). In the context of substance use disorders (SUD), a salient social identity may be that of a ‘substance user’, which often becomes dominant and negatively affects one's narrative identity and other valued roles.

Recovery, then, involves a re-authoring process in which this dominant identity is challenged, and alternative, more positive narratives are pursued. This process often entails the emergence of a recovery identity − a new or reconstructed social identity grounded in abstinence, agency, communion and future orientation (Dingle et al., 2015, McIntosh, 2002).

In this framework, narrative identity transformation and the adoption of a recovery-oriented social identity are deeply intertwined. Patients re-evaluate their former lives and selves (McWeegman, 2010), begin to tell more hopeful stories about who they are becoming (McIntosh, 2002, Singer et al., 2013, Singer, 2013), and gradually shift their sense of self away from the ‘addict’ identity towards a recovery-oriented narrative (Rowlands et al., 2019).

### 1.2 Narrative foreclosure

Transformation of one’s narrative identity requires a belief that one’s future is ‘open’ (Pickard, 2014), that new commitments and experiences in one’s future can substantially change one’s life-story (Bohlmeijer et al., 2011). In the recovery of SUDs, one can develop the belief in an open future by reinterpreting the past, so that meaningful change (McConnell & Snoek, 2018) or “new endings” (Singer, 2013, Singer et al., 2013) may become more plausible. When narrative reasoning fails and one cannot reinterpret aspects of one’s life-story, the resulting interpretative crisis is called “narrative foreclosure” (Freeman, 2000, Bruner, 1986). Narrative foreclosure (NF) entails a breakdown in narrative flexibility: past, present, and anticipated future are no longer meaningfully related or open to reinterpretation (McAdams, 1988, Sarbin, 1986, Bohlmeijer et al., 2014, Brandon et al., 2007), and one’s life story becomes constrained rather than evolving [21, 22]. NF can thus be understood as a foreclosure of narrative potential − a reduction in the capacity to revise the past or imagine an alternative future. It is suggested that transformation of one’s narrative identity will be involved in nearly all attempts at SUD-recovery (McConnell & Snoek, 2018). Considering how NF debilitates one’s capacity for narrative reasoning, it could be hypothesised that foreclosure makes it difficult to ‘rewrite’ one’s SUD-related ‘narrative identity’.

### 1.3 STUDY AIMS

While research findings to date are preliminary, they suggest that the evaluation of one’s self as communal and agentic (Rowlands et al., 2019) and the transformation of one’s narrative identity is a critical component of successful SUD-treatment (Dingle et al., 2015, McConnell and Snoek, 2018). How these findings relate to Cognitive Behavioural Therapy (CBT)-based SUD treatment has not been researched.

By challenging distorted thought patterns, CBT promotes the re-evaluation of past experiences in a way that fosters a more capable and resilient self-view. Additionally, CBT's focus on behavioural modification and skills training may enhance both perceived and actual agency, leading to self-narratives emphasising growth and the ability to overcome challenges. There is indirect evidence therefore, that CBT could positively affect one’s narrative identity. We choose to focus on CBT specifically, as it’s the primary treatment recommendation in clinical guidelines. As SUD relapse rates range between 40 % and 60 % within the first year post-treatment (Brandon et al., 2007, Dennis et al., 2005, Bradizza et al., 2006), it raises the question whether CBT adequately addresses the narrative work required to facilitate SUD-recovery. Furthermore, NF specifically could be an important factor in SUD-relapse post-treatment.

The present (explorative) study therefore, aims to answer three research questions: (1) ‘does CBT in a subgroup of SUD-patients contribute to decreased (narrative) foreclosure and increased evaluation of one’s self as communal and agentic post-treatment?’; (2) ‘how do the narrative themes of communion, agency and narrative foreclosure relate to SUD-treatment outcomes?’; and (3) ‘how are communion, agency and narrative foreclosure associated with relapse during the first 3 months after treatment?’.

## 2 Methods

### 2.1 Participants and procedure

The scientific ethical committee of the addiction treatment centre granted approval for the current study. We recruited participants from an outpatient clinic for adults with a Substance Use Disorder (SUD) in [anonymized], the Netherlands. We assigned patients to either individual or group CBT sessions for the treatment of SUDs based on their preferences. While this introduces the possibility of third-variable influences, we attempt to control for this within the results section. The treatment protocol begins with four sessions dedicated to building motivation, followed by sessions focused on changing dysfunctional thoughts, behaviours, skill-building, and relapse prevention. The CBT-based treatment consists of weekly sessions over a 12-week period. Each session lasted approximately 60 min, resulting in a total of up to 12 h of therapeutic contact time per participant. At the initiation of treatment, we invited patients to take part in this study. We provided information about confidentiality and the research's purpose, and obtained their consent to use their data and retrieve additional patient data from electronic records. Participants completed an informed consent form and, upon giving their written consent, filled out two additional self-report measures. Three months after completing treatment, these measures were administered again. A further three months later, the first author conducted a follow-up measure via phone. An a priori power analysis was conducted for a multiple linear regression with five predictors, using a two-tailed test, α = 0.05, power = 0.80, and an expected effect size of f^2^ = 0.10. This effect size was defined as the smallest effect size of interest (SESOI), representing the minimal association between narrative variables and treatment outcomes considered meaningful in this context. Based on these parameters, a minimum of 81 participants was required. Given that attrition is common within our treatment facility − and in addiction care more broadly − we anticipated a dropout rate of 25 %. The target sample size therefore, was set at 110 participants. Data collection was concluded once this number had been reached, regardless of the number of participants who completed all follow-up assessments. To minimise attrition, participants were contacted between sessions and received appointment reminders.A sensitivity power analysis was conducted to assess the minimum detectable effect sizes for each research question (see Section 3.2).

### 2.2 Measures

The “Agency and Communion Inventory” developed by Abele and colleagues (2016) is used to measure one’s identification with aspects of communion and agency. The scale consists of 20 items, presented in a bipolar format with a 5-point Likert scale. consists of 20 bipolar adjective pairs rated on a 5-point Likert scale, assessing both communion and agency. Example items include: “uncaring – caring” (communion) and “efficient – inefficient” (agency). In accordance with the first author and a small committee of 3 native Dutch and 2 native German speakers, the scale was translated from German to Dutch, back to German and a consensus-version was established. In the present study, both the ‘communion’ and ‘agency’ scales showed a high internal consistency with (both) Cronbach’s α = 0.80. This finding is consistent with internal consistency measures of the original instrument (resp. α = 0.80 & α = 0.77, (Abele et al., 2016)).

The Dutch Narrative Foreclosure Scale (NFS) is a 10-item self-report questionnaire assessing narrative foreclosure in relation to both the past and the future. Example items include: *“I have not done in life what I would have most liked to do”* (past) and *“I expect that new chapters will be added to my life story”* (future). Items are scored on a 4 point Likert scale, with higher scores reflecting a greater sense of foreclosure. The scale was previously validated by Bohlmeijer and colleagues (2014) in two samples of Dutch adults from the general population. In the present study, alpha coefficients are deemed ‘acceptable’ at α = 0.70 for both subscales.

Finally, behavioural outcomes are routinely measured with the Dutch version of the “Measurement of Addiction for Triage and Evaluation” (MATE (Schippers et al., 2011)) – as is custom with any patient seeking SUD-treatment in the Netherlands. The MATE includes an inventory of substance use in the last 30 days and includes the 21-item version of the Depression, Anxiety and Stress-Scale (DASS-21 (Schippers et al., 2011, de Beurs et al., 2001)). The MATE allowed us to include a measure for poly-substance use. The DASS-21 is a self-report measure which is rated on a 4-point Likert scale and is included as an outcome measure as emotional distress is often co-occurrent to SUD-use (Kluwe-Schiavon et al., 2020) and it is associated with continued SUD-use during and post-treatment (Greenfield et al., 2012). A representative item is: *“I felt that I was rather touchy”*. Internal consistency of the three separate factors is high (α = 0.94, α = 0.88 & α = 0.93 for the depression, anxiety and stress-scales respectively (de Beurs et al., 2001)). Psychometric evaluations however, have highlighted overlap between the depression, anxiety, and stress items, questioning the discriminant validity of the three subscales (Osman et al., 2012, Haynes et al., 1995). We therefore opted to use the total score as a global index of psychological distress, in line with recommendations for clinical research when subscale interpretation is not central to the hypotheses.

### 2.3 Statistical analyses

In line with the three research questions formulated in Section 1.3, our analyses addressed: (1) changes in narrative foreclosure and self-evaluation on agency and communion across treatment; (2) associations between baseline levels of these narrative themes and treatment outcomes; and (3) their relation to relapse within three months after treatment.

An overview of participant baseline characteristics is provided. As treatment drop-out is a frequent occurrence in addiction care, we used either an independent samples *t*-test or Mann-Whitney *U* test, testing for differences in all previously described measures between the group who dropped out of treatment and those who completed treatment. Then, to answer the first research question, we conducted linear mixed model (LMM) analyses using six models: one for each narrative theme and treatment outcome variable. Time was included as a factor in each model. Mean differences, F- and p-values are reported, as are Cohen’s d-values, as a measure of effect size (McGraw & Wong, 1996). The LMM analyses accommodate incomplete observations under the assumption of data missing at random (MAR). An overview of all experimental variables on all three timepoints is also provided and sample sizes, either means and standard deviations, or medians and interquartile ranges are reported (where applicable).

Two separate multiple linear regression analyses were conducted to answer the second research question. For these analyses, Substance-use frequency reduction and DASS-21 sum scores reduction (T0-T1) were used as dependent variables in the two models respectively. Following the approach of Grant and colleagues (2019), we employed 'univariable pre-screening' to reduce the set of independent variables to a smaller set, including them as 'predictor variables' in the two models when univariate analysis yielded a p-value of p < 0.15 between the predictor and dependent variable. We included narrative themes as independent variables. We also included the number of previous treatment attempts (McConnel, 2016), ‘age’ (Bohlmeijer et al., 2011), ‘gender’ (Holzhauer et al., 2020) and highest achieved education (e.g. Baptiste-Roberts and Hossain, 2018, Williams and Chang, 2000). Additionally ‘depressant use’ (i.e. alcohol, sedatives); ‘cannabis use’; ‘stimulant use’ (i.e. cocaine, amphetamine use) and ‘gambling’ are included as independent variables. These analyses were conducted on complete cases; no imputation was applied.

Then, using either backward or forward elimination (excluding independent variables when p > 0.15), multivariate regression models are constructed where subsequently non-significant variables were removed, one by one, until the total explained variance (R^2^) is reduced by more than 10 % when compared to the preliminary model. We employed the forward elimination method in analyses where there would be more than 1 independent variable per every 10 participants in the preliminary model. In other cases, we used a backwards elimination method.

The final research question considers the predictive value of baseline narrative themes in relation to SUD-use relapse at T2 (3 months post-treatment). A nominal variable ‘level of treatment outcome’ was constructed with two values: ‘0′, reflecting either ‘continued substance use or relapse’, and ‘1′, reflecting ‘abstinence or recovery’. As our study focused on short-term treatment effects, we used ‘abstinence’ as an indicator for (early) treatment success. Given the lack of consensus in the literature on how to define ‘relapse’ (Moe et al., 2022), we applied five different cut-off points for the ‘continued substance use or relapse’ group (0, 1, 2, 3, 4 or 5 days of substance use in the past 30 days, respectively). Binary logistic regression analyses were then conducted, including baseline narrative themes as independent variables while controlling for the previously mentioned covariates. These analyses were conducted on complete cases only, without imputation. Finally, odds ratios, confidence intervals and p-values are reported.

## 3 Results

### 3.1 Participants baseline characteristics

109 patients were asked to participate in the study. 107 patients gave consent and completed the first measures. 24.3 % of cases dropped out of treatment in the first 3 months. This finding is in line with attrition rates reported within our treatment facility. 36.7 % did complete treatment, but did not complete the second and third measure and 39 % completed all three measures. An independent samples *t*-test, did not find any significant differences on the baseline measures, comparing those who dropped out of treatment to those who completed all measures and no differences were found between individual or group therapy (data not shown). Of all participants, 72.9 % were male. The mean age was 39 years.

Most participants sought help with an alcohol related SUD, followed by a disorder in cannabis-use, cocaine and other substances. 72.0 % of participants participated in group-sessions of CBT, 28.0 % completed individual sessions of CBT in their treatment of SUDs. For further details, see Table 1.

**Table 1:** Baseline Participant Descriptive Statistics.

	Value (N(%))
Valid Observations	107
Gender	
male	78 (72.9 %)
female	29 (27.1 %)
Age (mean (SD))	39.20 (12.21)
Level of Education	
none completed	1 (0.9 %)
primary school	10 (9.3 %)
secondary education	46 (43 %)
tertiary education	43 (40.2 %)
graduate or higher	7 (6.5 %)
Occupation	
unemployed	28 (26.2 %)
employed	67 (62.6 %)
Study	6 (5.6 %)
(socially) organized activities	6 (5.6 %)
Intimate relationship	
no	49 (45.8 %)
yes	58 (54.2 %)
Previous Treatments (mean (SD))	0.70 (1.08)
Primary SUD	
alcohol	43 (40.2 %)
cannabis	30 (28.0 %)
opioid	1 (0.9 %)
cocaine	11 (10.3 %)
stimulants	8 (7.5 %)
XTC/MDMA	2 (1.9 %)
sedatives	3 (2.8 %)
gambling	9 (8.4 %)
SUD (years) median (IQR)	6.00 (3.00–11.00)
Substance use in last month (days) median (IQR)	21.00 (9.00–30.00)

Cells show observed frequency or means, unless otherwise specified. IQR = Interquartile Ranges (25th-75th percentile).

### 3.2 Narrative identity transformation

Table 2 shows the repeated measures within the present study at either both or all 3 time points (where applicable).Next, data retrieved from both Linear Mixed Models and Mann-Whitney U-tests, are displayed in Table 3. For the ‘SUD-use frequency’ model, we found a significant reduction in substance use, with findings indicating a large effect size (Cohen’s d = -0.93, p < 0.001)). We note that these analyses with data imputation as a means to correct for missing data, showed similar results. For the ‘DASS-21 sum score’ model, we also found a significant reduction in DASS-21 sum scores during treatment, with a moderate effect size (d = -0.42, p = 0.010). None of the four ‘narrative themes’ (communion, agency and narrative foreclosure (past and future)) showed a reliable or meaningful difference in LMMs. Mean differences for all these variables were less than 0.01, and p-values were not near statistical significance. See Section 3.2 for sensitivity analysis regarding the detectability of change in narrative themes.

**Table 2:** Overview of measures used to assess narrative themes, emotional distress, and substance use.

	T0	T1	T2
	M(SD) / Mdn (IQR)*	M(SD) / Mdn (IQR)*	M(SD) / Mdn (IQR)*
Valid N	N = 107	N = 41	N = 54
Substance use (days / month) median (IQR)	21.00 (9.0–30.0)	0.00 (0.0–8.0)	0.00 (0.0–10.0)
	4.09 (0.55)	4.11 (0.52)	−
AC-IN: Communion			
	2.8–5.0	2.9–5.0	−
	3.53 (0.60)	3.59 (0.64)	−
AC-IN: Agency			
	2.1 – 4.9	2.4–4.8	−
	2.53 (0.59)	2.41 (0.66)	−
NFS: Past			
	1.0–4.0	1.2–3.8	−
	1.50 (0.53)	1.53 (0.53)	−
NFS: Future			
	0.8–3.2	1.0–2.6	−
DASS-21 Sum Score Median (IQR)	38.0 (18.0–52.0)	18.0 (5.0–44.0)	−

Cells show mean, SD and range (lowest to highest observed values), or Median and Interquartile Ranges (25th-75th percentile). Abbreviations: DASS-21: Depression Anxiety Stress Scale; AC-IN: Agency & Communion Inventory; NFS: Narrative Foreclosure Scale.

**Table 3:** Pre- to post-treatment changes in narrative themes.

	Mean diff. T0-T1	F/Z	P-Value
*Mann Whitney U-Tests*			
SUD-use frequency (ranks)	43.49	−5.67	<0.001
DASS-21 sum score (ranks)	20.26	−2.57	0.010
*Linear Mixed Models*			
AC-IN: Communion	0.03	−	0.584
AC-IN: Agency	0.07	−	0.451
NFS: Past	0.08	−	0.714
NFS: Future	0.07	−	0.617

Abbreviations: DASS-21: Depression Anxiety Stress Scale; AC-IN: Agency & Communion Inventory; NFS: Narrative Foreclosure Scale.

Narrative Themes and Treatment Outcome.

In univariate linear models, we found that the primary substance used did not significantly relate to ‘SUD-use frequency reduction’ (no p-values lower than 0.15; data not shown). However, it had a large effect on DASS-21 sum score reduction during treatment (η2p = 0.29, p = 0.028). The primary substance used therefore, is included in the regression model, where DASS-21 sum score reduction is the dependent variable.

The data displayed in Table 4 show the parsimonious (multivariate) models of ‘SUD-use reduction’ and ‘DASS-21 sum score reduction’ during treatment. The first model showed that 15.1 % of variance in (absolute) SUD-use reduction could best be predicted by baseline ‘AC-IN: Communion’ and ‘NFS: Past’ scores, where higher scores were related to greater ‘SUD-Use Reduction’. We noted that the ‘communion’ measure is marginally significant (p = 0.053) and the ‘NFS: Past’ measure was not statistically significant. Finally, ‘NFS: Future’ showed an inverse relation with reduction in frequency of substance use, though this finding again, was insufficiently significant (p > 0.2).

**Table 4:** Predictive value of baseline narrative themes on treatment outcomes.

	SUD-Use Reduction		DASS-21: Sum Score Reduction	
	Multivariate Analysis Model R^2^: 0.151		Multivariate Analysis Model R^2^: 0.415	
	Un. β	P-value	Un. β	P-value
Intercept	−24.47	0.233	−44.99	0.175
SUD: gambling	NI	NI	−	−
SUD: cannabis	NI	NI	28.19	0<.001
SUD: stimulants	NI	NI	−	−
AC-IN Communion (T0)	7.65	0.052	17.26	0.017
AC-IN Agency (T0)	−	−	−	−
NFS: Past (T0)	5.35	0.156	−	−
NFS: Future (T0)	−4.97	0.237	−18.50	0.015

Abbreviations: Agency & Communion Inventory (AC-IN); Narrative Foreclosure Scale (NFS); NI = Not Included (in model).

Reduction in ‘DASS-21 sum scores’, was best predicted by the second model displayed in Table 4. The model explained up to 41.5 % of variance in the dependent variable. DASS-21 sum score reduction could best be predicted by baseline ‘cannabis use’, ‘AC-IN: Communion’ and ‘NFS: Future’. ‘NFS: Future’ was negatively related to DASS-21 sum score reduction during treatment.

Participants who used cannabis at baseline, would show a greater reduction in DASS-21 sum scores during treatment, when compared to participants who sought help with another primary substance used. Greater baseline ‘communion’ also appeared to be related to greater DASS-21 sum score reduction.

For statistical power considerations relevant to these models, see Section 3.2.

### 3.3 Narrative foreclosure and SUD-Relapse

Our sample yielded substance use frequency data on T2 for 53 participants. Using a cut-off value of ≤ 4 days of substance use in the past 30 days, 34.0 % of cases classified as ‘continued substance use or relapse’ (n = 18), while 66.0 % classified as ‘abstinence or recovery’ (n = 35). Other than baseline narrative themes, only gender showed a univariate relation to either ‘level of treatment outcome’ (G(1) = 4.53, p = 0.033).

The regression analysis displayed in Table 5 showed that both baseline ‘AC-IN: Communion’ and ‘NFS: Future’ scores, were significantly related to the level of treatment outcome and explained up to 34.9 % of variance within this dependent variable. ‘AC-IN: Communion’ specifically, showed a moderate positive relation to level of treatment outcome (Exp(B) = 5.98, p = 0.016), while ‘NFS: Future’ showed a moderate inverse relation to the dependent variable (Exp(B) = 0.021p = 0.031).

**Table 5:** Associations between baseline narrative themes and relapse within 3 months post-treatment.

Level of Treatment Outcome (0/1)		
Multivariate Analysis Model R^2^: 0.349		
	Exp(B) (95 % CI)	P-value
Constant	0.02	0.190
Gender	−	−
AC-IN Communion (T0)	5.98 (1.40–25.54)	0.016
AC-IN Agency (T0)	−	−
NFS: Past (T0)	−	−
NFS: Future (T0)	0.21 (0.05-0.87)	0.031

Abbreviations: Agency & Communion Inventory (AC-IN); Narrative Foreclosure Scale (NFS).

These findings largely persisted when we lowered the cut-off value for the’recovery group’. When a cut-off value of 0 or 5 was used (suggesting either 0 or ≤ 5 days of substance use at T2), both communion and NFS: Future together, explained 20.8 % to 32.2 % of variance in level of treatment outcome respectively, with NFS: Future losing some statistical significance (Exp(B) = 0.35 to 0.30 and p = 0.105 to 0.076 respectively). These analyses were also repeated with cut-off values 1, 2, 3, showing no meaningful difference (data not shown). The sensitivity of these results is further evaluated below.

### 3.4 Sensitivity analysis

To assess the robustness of key findings, a sensitivity power analysis was conducted based on the sample sizes used across models.

Only moderate or larger effects (f^2^ ≥ 0.19) could be reliably detected with regards to the first two research questions. The absence of significant changes in narrative themes during treatment, or marginal findings (e.g., communion, p = 0.053), should therefore be interpreted with caution.

For logistic regression predicting relapse (n = 53), detectable odds ratios were approximately ≥ 3.0. Observed effects for Communion and NFS: Future were consistent across varying cut-off definitions, but smaller effects may have gone undetected.

Overall, the study was sufficiently powered to detect moderate-to-large effects, yet underpowered for detecting more subtle associations − especially for exploratory predictors such as narrative constructs.

## 4 Discussion

The study’s primary objective was to explore the extent to which CBT-based SUD-treatment supports the transformation of one’s narrative identity (as expressed in both ‘communion’ and ‘agency’ (Abele et al., 2016)), presumed to be a critical factor in successful SUD-recovery (Dingle et al., 2015, McConnel & Snoek, 2018). Furthermore, the study aimed to assess if CBT-based SUD-treatment supports the resolution of narrative foreclosure (NF) and the role of all these narrative themes in relation to CBT-based SUD-treatment outcomes at both CBT-protocol end and follow-up.

### 4.1 Narrative identity transformation

Corresponding with previous research (Carrol & Onken, 2005), we found large effects of CBT-based SUD-treatment on emotional complaints and frequency of substance use. However, our findings indicate that the treatment did not significantly impact the transformation of narrative identity or the resolution of narrative foreclosure. These findings could not be explained by misrepresentation of the sample due to drop-out and suggest that CBT based SUD-treatment may insufficiently aid the transformation of one’s narrative identity in terms of communion, agency and the resolution of narrative foreclosure. Our findings therefore invite a nuanced reflection on the (long-term) effectiveness and sustainability of CBT-based SUD treatment outcomes, on which we will expand below.

### 4.2 Narrative themes and treatment outcome

Our study demonstrates how specific narrative themes are associated with treatment outcomes, as measured from baseline to the end of the CBT protocol. Both baseline communion and future-oriented narrative foreclosure (NF: Future) showed statistically significant associations with outcome indicators, though in opposite directions. Higher levels of communion were linked to greater reductions in substance use and emotional complaints. In contrast, higher levels of NF: Future were associated with poorer treatment outcomes on both these measures.

However, when considering the reduction in the frequency of substance use as a continuous outcome, the results were less robust. Baseline communion showed only a marginally significant association with reductions in substance use frequency, indicating that this aspect of treatment response was harder to predict based on narrative factors alone.

Interestingly, past-oriented narrative foreclosure (NF: Past) showed a large − though statistically non-significant- positive association with substance use reduction during treatment. This may suggest that certain emotions linked to past-directed NF, such as regret or depressive reappraisal, could in some cases enhance motivation for change. While speculative, this points to a potentially constructive role for past-directed narrative engagement, and warrants further exploration in future research.

### 4.3 Narrative themes and relapse

To provide context for our findings, we feel it's essential to differentiate between abstinence and recovery. As we focussed on treatment outcome in the short term, we used ‘abstinence’ as an indicator of (early) treatment success. While it does not express the complexity of recovery, it serves as a critical early indicator of the recovery process.

As with their relation to treatment outcome, baseline communion and NF: Future both show moderate to large effects on post-treatment relapse rates. These two factors alone, explain up to a quarter of variance in relapse rates at follow-up. Given the lack of consensus on defining 'relapse' in SUD research, we employed varied criteria to support our findings. Notably, baseline 'communion' predicts treatment outcomes, aligning with prior research (Rowlands et al., 2019, McWeegman, 2010) and reflecting the emphasis within 12-step programs, for example, on strengthening communal bonds. This underscores the importance of communion in shaping recovery trajectories. Next, we contribute to existing literature by demonstrating that higher levels of NF: Future adversely affect CBT-based SUD recovery, both at treatment end and follow-up. Individuals with greater narrative foreclosure to their future narratives would tend to benefit less from treatment.

### 4.4 Contrasts with previous research

We could not substantiate the claim made in previous studies (e.g. Adler, 2012, Chen, 2018) or by Rowlands and colleagues (2019) that “identity transformation in recovery [involves the] twin processes of agency and communion”, as the’agency’ construct showed no relation to any treatment outcome in the current sample. This contrasts our own expectation based on other sources in recent literature: SUDs negatively impact numerous agentic themes (e.g. self-worth (Van Erp and Van der Meulen, 2017, Van Boekel, 2015); self-esteem and sense of empowerment (Livingston & Boyd, 2010)). Although our findings do not support a direct relationship between agency and treatment outcome, we considered the possibility that agency may mediate the relation between NF: Future and recovery. This hypothesis is theoretically plausible, given known associations of NF: Future with low extraversion, low ego-integrity, and diminished hope (Bohlmeijer et al., 2014), all of which are conceptually linked to agentic functioning (Abele et al., 2016). While we conducted an exploratory analysis to explore this idea post hoc, the absence of a pre-specified hypothesis, combined with limited statistical power, led us to omit the results from the current manuscript. We encourage future research to examine this model with appropriate sample sizes and pre-registered designs.

### 4.5 Limitations and recommendations

We recognize that the reliance on convenience sampling and the lack of follow-up data for participants who dropped out or did not complete treatment present important limitations. Although baseline similarities between groups (e.g., completers vs. non-completers) mitigate some concerns, these limitations restrict the generalizability of our findings — particularly with regard to long-term outcomes. In addition, the attrition rate was considerably higher than anticipated: while our a priori power analysis accounted for a 25 % dropout rate, only 39 % of participants completed all three assessments. This discrepancy substantially reduced the statistical power for longitudinal analyses, increasing the risk of Type II errors and limiting the precision of effect size estimates. Although linear mixed models allowed us to retain partially complete cases, the small number of full cases may compromise the robustness of certain inferences. Finally, we considered the potential influence of treatment modality. However, outcomes did not differ significantly between individual and group-based CBT, suggesting that narrative processes may operate similarly across formats. Despite these limitations, the sample remains representative of individuals seeking SUD treatment at Dutch outpatient clinics, supporting the contextual relevance of the findings.

Given CBT’s status as the preferred treatment modality for SUDs in the Netherlands (GGZ Standards, 2017), our findings offer insight into how narrative structures and themes relate to short-term recovery. In particular, this study highlights the predictive role of future-directed narrative foreclosure (NF: Future). While CBT may effectively reduce substance use and emotional distress in the short term, its impact appears to depend on favourable narrative conditions at treatment onset — such as low NF: Future and high levels of communion. This suggests that CBT, though beneficial for many, may fall short for clients whose self-narratives are rigid or foreclosed. This may, in part, reflect the relatively brief duration of treatment, and the fact that CBT for SUD was not designed to explicitly target self-narrative themes. Clients with favourable narrative profiles may benefit substantially from CBT alone, while those with elevated NF or diminished communion might require supplementary interventions that directly target narrative identity.

Therefore, we echo recent calls to integrate narrative-focused elements into CBT protocols for SUDs (McConnel & Snoek, 2018). Although beyond the scope of the present study, future research might also explore how our findings generalise to other behavioural approaches, such as motivational interviewing or contingency management.

While our focus on short-term treatment effects, using abstinence as an early indicator of success, offers valuable insights, it is important to note that initial abstinence may not reliably predict long-term recovery outcomes. Thus, the generalizability of our findings to long-term recovery remains limited. Nonetheless, these results lay the groundwork for future research and highlight the potential benefit of integrating narrative-focused interventions into SUD treatment.

With regards to future directed NF, we assume overlap with the ‘demoralisation’ construct (Kissane et al., 2001) and if correct, SUD-based CBT could perhaps be enhanced by integrating CBT principles for the resolution of demoralisation (Beck et al., 2008). Further research could substantiate and expand on our findings and ideas, preferably with a greater sample size and by further testing the validity of our translated version of the AC-IN (Abele et al., 2016). Finally, we call for the establishment of a clear, standardised definition of 'recovery' and 'relapse,' as the lack of such definitions currently limits the interpretability of studies like ours and complicates direct comparisons between research outcomes.

## Declaration of competing interest

The authors declare that they have no known competing financial interests or personal relationships that could have appeared to influence the work reported in this paper.

## Data Availability

Data will be made available on request.
